# Effects of Electroacupuncture Therapy and Cognitive Behavioral Therapy in Chronic Insomnia: A Randomized Controlled Study

**DOI:** 10.1155/2020/5630130

**Published:** 2020-03-19

**Authors:** Jia Xing, Xi Wu, Hongxia Liu, Jialin Wang, Siyuan Jiang, Aileen Lozada, Yifan Wang

**Affiliations:** ^1^Rehabilitation Department and Acupuncture and Moxibustion Department, Dongfang Hospital, Beijing University of Chinese Medicine, 6 First Zone, Fangxingyuan, Fangzhuang, Fengtai District, Beijing 100078, China; ^2^Psychological Department of Guangnei Community Health Service Center, No. 49, Five Alleyway of Jiaochang, Guangnei Street, Xicheng District, Beijing 100053, China; ^3^Yin Yang Balance Center, 3400 Coral Way Suite 400, Miami, FL, USA; ^4^Beijing University of Chinese Medicine, No. 11, East North Third Ring Road, Chaoyang District, Beijing 100029, China

## Abstract

**Objective:**

To evaluate the efficacy of spirit-regulating electroacupuncture (EA), cognitive behavioral therapy (CBT), and combined EA/CBT to treat insomnia.

**Methods:**

In this prospective clinical study, patients were randomly assigned to receive EA, CBT, or EA/CBT. Outcomes were assessed using PSQI, ISI, ESS, DBAS-16, HAM-A, and HAM-D at two- and four-week follow-up.

**Results:**

Overall, the PSQI and ISI scores decreased after treatment in all three groups. At two-week follow-up, the EA/CBT group obtained lower PSQI and ISI scores than the EA group. The daytime functional factor score in the EA group was lower than that of the CBT group after the second week of treatment, and the EA/CBT PSQI score was lower than that of the CBT group on the second week of follow-up. In comparison with baseline, the EA group had a decreased ESS score after the second and fourth weeks of treatment, while the ESS score increased in the CBT group after the fourth week of treatment. ESS scores were unchanged following treatment in the EA/CBT group. After the second and fourth weeks of treatment, the ESS scores from the CBT group were higher than the EA group. The DBAS-16 decreased in the CBT and EA/CTB groups, while the EA group had a higher DBAS-16 score. In all three groups, HAM-A and HAM-D scores decreased after treatment; the EA/CBT HAM-A and HAM-D scores were lower than the other two groups.

**Conclusion:**

1. Spirit-regulating EA therapy is effective. In terms of improving sleep quality and mood, EA has the same effect as CBT and can improve daytime function earlier. 2. The curative effect of the EA/CBT group lasts longer than that of the EA group, and EA/CBT is better at improving daytime function compared to CBT alone and better at improving mood compared to CBT or EA alone.

## 1. Introduction

Insomnia is a common clinical symptom. In the past decade, the International Classification of Sleep Disorders (ICSD-3) [1] and the Diagnostic and Statistical Manual (DSM-V) [2] have disregarded the etiology classification of insomnia, focusing on the classification of disease. Chronic insomnia causes not only clinical pain, but also damage to social, occupational, educational, academic, behavioral, and other important functions. Insomnia also consumes a large amount of medical resources [3], causing losses of social and economic property [4]. According to an epidemiological survey, chronic insomnia affects 6–10% of the population [5]. Insomnia has become an urgent health problem and has received unprecedented attention.

Drug treatment for chronic insomnia is dominated by gamma-aminobutyric acid (GABA), including benzodiazepines and non-benzodiazepines. However, their clinical applications due to tolerance, dependence, and addiction are limited; and there is insufficient evidence to support their long-term efficacy [6]. Although the new generation of sedative and hypnotic drugs has moderated side effects, they often improve symptoms without completely curing insomnia. Nondrug therapies include psychological behavioral therapy, as well as complementary and alternative medicine. Cognitive behavioral therapy (CBT) is the most important treatment in the field of psychological behavioral therapy. CBT for chronic insomnia (CBT-I) can change negative thinking and beliefs or bad cognition and behavior that affects sleep through certain methods and break the conditions to awaken and readvise new good sleeping habits. There is sufficient clinical and experimental evidence demonstrating that CBT-I is highly effective, has no side effects, and has long-term benefits over drugs [7–10]. The American College of Physicians (ACP) recommends CBT-I as an initial treatment for chronic insomnia [11]. Although CBT-I is recognized by the latest international guidelines and recommended as the first-line treatment for chronic insomnia, its clinical implementation is still subject to multiple factors, such as the lack of professional therapists, long cycle, high cost, and occasional poor or failed efficacy [12].

Acupuncture for chronic insomnia is a complementary treatment. Chinese medicine focuses on the combination of body and spirit and believes that the onset of insomnia is closely related to the dysfunction of “Spirit.” Therefore, acupuncture treatment attaches great importance to regulating the spirit. Acupuncture is a common method used in China to treat chronic insomnia, but it has not been universally recognized [13] and requires more evidence-based medical research. While acupuncture is often performed during the day, previous acupuncture studies have focused on its impact on sleep quality. The improvement of daytime function is worth further exploration. Daytime function includes daytime sleepiness, alertness, physical well-being, and ability to concentrate in the daytime. The sleep-wake cycle disorder of chronic insomnia can cause daytime dysfunction and contribute to home, traffic, and work-related accidents [14]. Drowsiness during driving is estimated to be the cause of 2–16% of car accidents [15]. The evaluation of daytime function, in addition to subjective methods such as scales and questionnaires [16], includes objective methods such as multiple sleep latency, maintenance of wakefulness, and driving simulator tests.

Based on the literature regarding CBT and acupuncture for treatment of insomnia, we designed a randomized, controlled clinical study to investigate the intervention effects of electroacupuncture (EA), CBT, or EA combined with CBT on sleep quality, daytime function, sleep cognition, and emotional state in chronic insomnia.

## 2. Methods

### 2.1. Design

We conducted a prospective randomized controlled clinical study to compare the efficacy of EA, CBT, and EA combined with CBT (EA/CBT) treatment for chronic insomnia. Efficacy assessments were performed at baseline, after two weeks of treatment, after four weeks of treatment, and in the second week following the completion of treatment. This clinical study complied with the Declaration of Helsinki (Edinburgh Edition 2000) and was approved by the Ethics Committee of the Dongfang Hospital of Beijing University of Chinese Medicine (JDF-IRB-2015031901).

### 2.2. Participants

The subjects were recruited from outpatients and advertisements in Dongfang Hospital of Beijing University of Chinese Medicine (May 2015–February 2018).

Inclusion criteria include the following: (1) meeting the insomnia diagnostic criteria of DSM-V; (2) complying with the disease standard of “China Guidelines for the Prevention and Treatment of Insomnia” [17] (the course of disease has been longer than six months); (3) not using or discontinuation of psychotropic drugs such as antidepressants in the past month; (4) absence of serious physical illness; (5) cooperating with group therapy and acupuncture treatment and volunteering to participate in this study, including a signed informed consent; and (6) cooperating with the completion of the scale assessment.

Exclusion criteria include the following: (1) being diagnosed with a severe mental illness or having suicidal tendency; (2) abuse or addiction of drugs or other substances (including alcohol, tobacco, caffeine, sedatives, and psychoactive substances.); (3) previous or current epilepsy, mania, bipolar disorder, or history of abnormal sleep; (4) respiratory-related sleep disorders found by polysomnography; and (5) adding any medications that improve sleep (including Chinese medicines, melatonin, or health supplements that have a calming effect) during study treatment.

Subjects who met the inclusion criteria were randomly assigned to EA, CBT, or EA/CBT treatment after signing an informed consent and evaluation by the designated assessor.

### 2.3. Sample Size

The sample size was estimated based on the Pittsburgh Sleep Quality Index (PSQI) [18] total score, assuming that the treatment group can reduce the total PSQI score by 18% compared to the baseline. Refer to the sample size estimation formula for continuous variables (note: X1 and X2 are the expected mean values of the treatment group and the control group; *σ* is the standard deviation of the control group). The standard deviation is 15% of the mean of the control group, that is, *α* = 0.05, *β* = 0.1, *f* (*α*, *β*) = 10.5, and then *m* = 30.24, about 30 cases per group. Assuming a dropout rate of 20%, 36 cases per group were selected to achieve statistical significance. The total sample size was 90 cases and the random number generated was 108.

### 2.4. Randomization and Allocation

SAS® software (Cary, NC, USA) was used to generate random numbers, and the patients were randomly assigned to EA, CBT, or EA/CBT according to a ratio of 1 : 1 : 1. The randomization allocation assignment was kept in an opaque envelope which the assistants sequentially opened according to the order in which the patient was screened.

### 2.5. Design Methods for Researcher Responsibility Isolation Blindness and Researcher's Qualification Resume

The therapist and psychologist who participated in the study provided a qualification resume. The acupuncture therapist and psychologist treated the subjects independently in separate spaces according to the study's intervention plan, without interfering with each other's treatment process, and without participating in the evaluation of the treatment efficacy of any subject. A psychological assessor with relevant qualifications completed the scale assessment in the psychological assessment room. The psychological assessor did not participate in the screening and treatment of participants.

### 2.6. Intervention

This study designed and reported acupuncture clinical trial interventions in accordance with the STRICTA [19] inventory criteria.

#### 2.6.1. EA Group

The acupuncture points used were DU-20, EX-HN1, EX-HN22, SP-6, HT-7, PC-6, BL-62, and KI-6. All the acupoints were inserted bilaterally except for DU-20 and EX-HN1. Yunlong brand sterile acupuncture needles 0.35 mm × 40 mm (Wujiang Yunlong Medical Instrument Co., Ltd.) were used. The degree of insertion for DU-20 and EX-HN1 was decreased by 0.8 inches; EX-HN22, SP-6, and PC-6 were all inserted one inch deep, while HT-7, BL-62, and KI-6 were inserted 0.5 inches deep. After the needle was inserted, the patient could feel mild numbness, swelling, and a stabbing pain [20]. DU-20 and EX-HN1 were connected to the positive and negative poles of the electric wire. We used sparse and dense waves, two Hz sparse waves, and 100 Hz dense waves, where the intensity reached the needle handle micro fibrillation (EA instrument YINGDI KWD-808I).

The course of treatment was four weeks. A total of 12 acupuncture treatments with a frequency of three times per week were administered, 30 minutes at a time; the interval between weekly treatments was one or two days. Each treatment was scheduled by the subject and the acupuncturist. Instead of discussing sleep with the acupuncturist, patients can talk about acupuncture treatments, such as acupuncture points in the treatment room.

#### 2.6.2. CBT Group

*(1) Implementation Mode of CBT Treatment*. The team was led by a professional psychotherapist and a clinician. Therapy occurred in a closed group of 8–10 people. Each session was 90–120 minutes, once a week for a total of four sessions.

*(2) Activity Content of CBT Treatment*. This study utilized the insomnia CBT standard program, combined with the actual situation of the research unit and the pretest process, with an independently designed CBT treatment plan for this study. That included four activities, each session containing at least one technique. In addition, participants completed a sleep diary and homework during the treatment.

The first session included circular self-introduction, discussion of treatment expectations, health education regarding normal sleep and the causes of insomnia, and meditation relaxation training with guiding words.

The second session included sleep hygiene education, sleep restriction therapy, homework and sleep diary review, developing behavioral therapy for the next week, and abdominal breathing relaxation training.

The third session included stimulus control therapy, learning to adjust sleep beliefs and reducing interference with sleep, homework review, adjusting behavioral treatment programs, and progressive muscle relaxation.

The fourth session included discussion regarding beliefs that affect sleep (homework from the previous session), discussing the implementation of sleep-aware cognitive behavioral skills, how to deal with the recurrence of sleep problems, and further adjusting behavioral treatment programs to maintain sleep efficiency.

*(3) Handling of Closed Group Privacy and Tightness*. Therapy sessions occurred at a fixed time at a fixed, quiet space without interruptions. Participants read the “Binding Book” in order to ensure the privacy of group therapy. During each session, “ice-breaking” activities occurred to promote group cohesion (it is a way of repeating the introduction of partners to complete the interactive introduction, which is different from the traditional way of introducing only their names),“angels in the world, angel gifts, angel disclosure (it is a small design. Everyone in every group is an angel and also a person guarded by an angel. At the end of the group's first activity, by drawing lots in turn, everyone will draw a name. This person is the angel he will guard during the whole treatment period, but he must keep it as a secret and cannot say the name of the person he guards. In the latter activities, he will learn about the man he is guarding through the exchange of information in the group and prepare gifts in each activity and give them to the therapist in the next activity. The therapist will send the gifts in turn at the beginning of the group activities. In the last group activity, there is a link; everyone takes turns guessing who is guarding them, hugs or shakes hands when they guess right, and thanks him for his company and guardianship. It can continue to guess or directly reveal angels and then express gratitude)”, “mood thermometers (it is a process of quantifying one's own emotions before each activity, giving a scale like a thermometer, and explaining them so that they can better understand each other's emotional state at this moment)”, “wearing high hats” (giving high praise), “graduation feelings” (before the last activity, everyone writes an impression of participating in the whole group CBT and reads it out in the last activity), and other small links were included to ensure the closeness of each group and the compliance of the subjects.

#### 2.6.3. EA/CBT Group

The protocol for EA treatment and the protocol for CBT treatment outlined above were followed for the EA/CBT group, including sessions.

### 2.7. Quality Control

All participating researchers, including acupuncturists, CBT therapists, and efficacy evaluators, had more than 10 years of professional work experience. In addition, protocol training was conducted prior to the start of the study to ensure treatment and evaluator consistency. The training included diagnostic criteria, inclusion and exclusion criteria, acupoint location, manipulation techniques, implementation of CBT, and completion of case report forms (CRFs). Regular monitoring was performed during the study to ensure the accuracy and quality of the study data.

### 2.8. Outcome Measures

PSQI, Insomnia Severity Index (ISI) [21], and Epworth Sleepiness Scale (ESS) [22] were administered at baseline, following two and four weeks of treatment, and two weeks after the completion of treatment. Dysfunctional Beliefs and Attitudes about Sleep (DBAS-16) [23], Hamilton Anxiety Scale (HAM-A), and Hamilton Rating Scale for Depression (HAM-D) were administered at baseline and at the end of the treatment period (four weeks).

1. PSQI measures sleep improvement using seven component scores and the total score.
2. ISI determines the severity of insomnia according to the total score of observation points.
3. ESS determines the degree of daytime sleepiness according to total score at observation time.
4. Dysfunctional Beliefs and Attitudes about Sleep (DBAS-16, Rev. 2007 Morin, Likert score) measures sleep-related cognition.
5. HAM-A and HAM-D (computer software version 1.07TX developed by Beijing Heisman) were used to evaluate the degree of anxiety and depression in patients before and after treatment.

### 2.9. Statistical Analysis

Data was entered using Microsoft Office Excel 2003 software. Enumeration data such as demographic data were tested by *χ*2 test and Cochran-Mantel-Haenszel (CMH) test. Outcome measurement data such as PSQI, ISI, ESS, DBAS-16, HAM-A, and HAM-D were tested by variance analysis, *χ*2 test, F test, and Kruskal-Wallis test. The study conducted an intentional analysis of full analysis set (FAS) and pre-protocol set (PPS). Student's *t*-test was used for comparison between groups. *P* < 0.05 was considered to be statistically significant. In order to avoid false positive errors of the nonparametric Kruskal-Wallis test of pairwise comparison, *P* < 0.0167 was considered to be statistically significant. The Bonferroni correction is used here.

## 3. Results

### 3.1. Study Population

**Figure 1:**
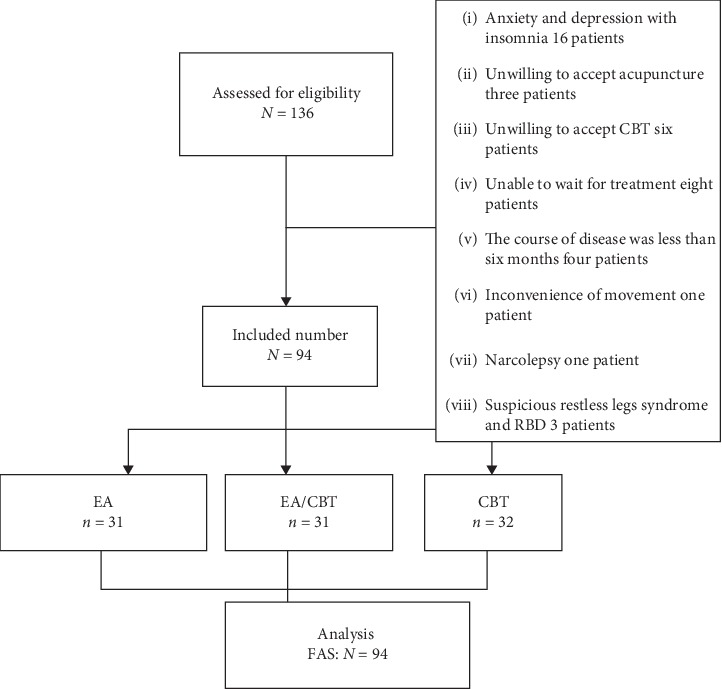
Trial profile.

A total of 136 patients were screened, 94 patients were eventually included, and three patients dropped out. 31 participants were randomized to EA; one participant dropped out; 32 participants were randomized to CBT, two participants dropped out; and 31 participants were randomized to EA/CBT, no one dropped out of this group. A total of 91 patients completed the study (Figure 1).

Gender, age, marital status, length of insomnia, education level, use of sedative sleeping pills, and pretreatment questionnaire scores were compared between the three groups, with no statistically significant difference (*P* > 0.05) found (Table 1).

### 3.2. Outcome Measurements

#### 3.2.1. Sleep Measure (Figure 2)

PSQI total score: compared with the baseline, the three groups showed statistically significant differences during the treatment and follow-up after treatment completion. After the second and fourth weeks of treatment, there was no statistical difference among the three groups; in the second week of follow-up, the EA/CBT group was lower than the EA group (*P* < 0.0167). The sleep quality factor scores of the PSQI in the combined group were lower than those in the EA group in the second week of follow-up (*P* < 0.0167). The sleep latency factor was lower in the EA group (*P* < 0.0167) after the fourth week of treatment and in the second week after follow-up. There were no statistical differences in the other factors among the groups. ISI total score: compared with the baseline, the three groups showed statistically significant differences in the observation time and follow-up time after treatment. There was no significant difference in ISI scores among the three groups after two weeks and four weeks of treatment; there was a statistical difference between the three groups in the second week of follow-up (*P* < 0.05), and the EA/CBT group was lower than the EA group (*P* < 0.0167).

#### 3.2.2. Daytime Functioning

The PSQI daytime dysfunction component was lower in the EA group than in the CBT group after two weeks of treatment (*P* < 0.0167), and the EA/CBT group was lower than the CBT group (*P* < 0.0167) in the second week of follow-up.

**Figure 3:**
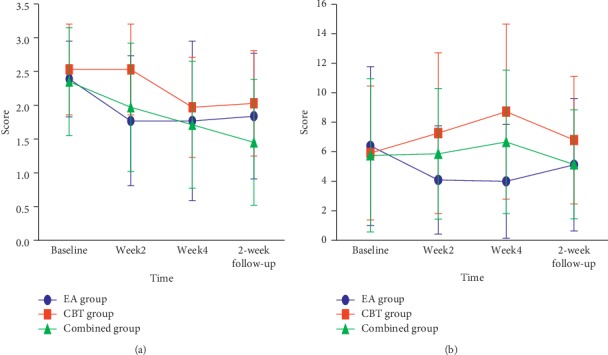
Change in daytime dysfunction factor of PSQI and ESS score at different times. (a) Daytime dysfunction factor. (b) ESS

ESS total score: compared with the baseline, the EA group ESS score decreased after the second week and the fourth week of treatment, and the CBT group ESS score increased after the fourth week of treatment. There was no change in the EA/CBT group. After the second and fourth weeks of treatment, there was a statistically significant difference in the total ESS score among the three groups (*P* < 0.05, *P* < 0.01), which was higher in the CBT group than in the EA group (*P* < 0.0167) (Figure 3).

#### 3.2.3. Sleep Cognition

DBAS-16 total score: compared with the baseline, the CBT group and the combined group DBAS-16 scores decreased (*P* < 0.01). There were differences among the three groups (*P* < 0.05); the EA group DBAS-16 score was higher than the CBT and EA/CBT groups (*P* < 0.0167) (Table 2).

#### 3.2.4. Emotion Assessment

Compared with the baseline, the HAM-A total score (Table 3) decreased in all three groups (*P* < 0.01). There was a difference among the groups (*P* < 0.05); the EA and CBT groups had higher HAM-A scores than the EA/CBT group (*P* < 0.0167). The total scores of HAM-D (Table 4) were lower in the three groups (*P* < 0.01). There were differences in HAM-D scores among the groups (*P* < 0.05). The EA and CBT groups' HAM-D scores were higher than the EA/CBT group (*P* < 0.0167).

### 3.3. Adverse Events

After EA treatment, there were three cases of bleeding after needle insertion and two cases of subcutaneous bruising. To treat bleeding, a cotton swab was used to apply pressure when the needle was removed. Bleeding resolved in all three cases without the need for any further treatment. After CBT treatment, there were 19 cases of drowsiness, five cases of dizziness and headache, and two cases of blood pressure fluctuation. The treatment for those patients was an increase in relaxation training and avoidance of strenuous exercise. There were no patients who fell due to adverse reactions to CBT treatment.

## 4. Discussion

Because of the periodicity and schedule, the follow-up period of this study is only 2 weeks, so only the short-term efficacy of the treatments is discussed preliminarily.

### 4.1. Spirit-Regulating EA Therapy

PSQI and ISI scores showed a dynamic decline between baseline and treatment. This indicates that spirit-regulating EA therapy can effectively improve the subjective sleep quality and severity of chronic insomnia in patients. Compared with the CBT group, EA was equally effective in terms of improving the insomnia severity index, sleep quality, sleep latency, sleep duration, sleep disturbance, and sleep efficiency during the overall intervention course and follow-up period. Comparing the EA group with the CBT group, the effect on the overall drug reduction was equivalent, but the EA treatment was able to reduce sedative use earlier in the treatment stage compared to CBT. The delay in CBT efficacy and corresponding reduction in sedative use may be related to the CBT treatment protocol. In the first week, only relaxation meditation was carried out, sleep hygiene education was taught in the second week, and in the third week cognitive discussion and correction on the use of sedative hypnotic drugs were conducted; the use of sedative hypnotic drugs was significantly reduced in the late treatment of the CBT group. Overall, treatment efficacy was not significantly different between the EA and CBT groups.

PSQI and ESS measure the severity of daytime dysfunction related to insomnia. The PSQI and ESS trends consistently suggest that, in the early treatment, EA can better improve the daytime function of chronic insomnia patients when compared to the CBT group, including the reduction of drowsiness and sleepiness. These results may be related to the effects of mild to moderate sleep deprivation caused by sleep restriction therapy (SRT), which was initiated in the second week of CBT treatment. SRT gradually adjusts as the patient's sleep efficiency improves. Therefore, as the treatment time is prolonged, the degree of drowsiness and sleepiness decreased accordingly. By the time of follow-up, there was no significant difference between the EA and CBT groups.

The DBAS-16 can reliably reflect the unreasonable beliefs and cognition associated with sleep in insomnia patients. In recent years, this has been widely used in various forms of CBT clinical research for insomnia [24, 25]. Previous studies have confirmed that targeted corrections about sleep beliefs and attitudes can improve sleep quality [26]. In our study, the total scores of DBAS-16 decreased after treatment in the CBT group and the EA/CBT group, suggesting that cognitive impairment was reduced, while the EA group showed no significant changes. There were also significant differences between the groups after treatment. One of the techniques of CBT treatment is the identification and correction of poor sleep cognition, thus indicating the success of the implementation of CBT treatment in this study. EA treatment, on the other hand, does not correct patients' misperceptions. The persistence of sleep efficacy is related to the improvement of sleep cognition [27]. This also explains the slight rebound phenomenon in PSQI and ISI scores after the EA treatment. Therefore, although the efficacy of EA and CBT treatments is equivalent, the mechanism of improvement may be different. This hypothesis requires further research to verify.

A longitudinal study found that insomnia and emotional state are closely related [28]. Chronic insomnia is often associated with mental symptoms such as anxiety and depression; studies [29, 30] also indicate that insomnia is a predictor of mental illness. Insomnia together with anxiety and depression are related to the monoaminergic transmitter system in the biology pathogenesis. It has been confirmed that the 5-HT_2A_ and 5-HT_2C_ receptors have a direct effect on sleep [31]. Although this study excluded patients with depressive episodes or anxiety disorders associated with insomnia, chronic insomnia associated with anxiety and depression is extremely common in clinical work. The results of this study suggest that both EA and CBT treatments can improve anxiety and depression in patients with chronic insomnia, and the effect is equivalent, while the combination of the two is more effective.

### 4.2. EA Combined with CBT Therapy

In this study, the PSQI total scores and the various factor scores, ISI and ESS total scores, of the three groups were observed at different periods. The statistical results indicate that combined EA/CBT therapy is effective in improving nighttime sleep quality and daytime function. EA/CBT was superior to EA in the improvement of sleep latency factor. During the follow-up period, the improvement of the EA/CBT group in terms of sleep quality, sleep latency, and sleep severity index was significantly better than the single EA group. EA/CBT treatment was also better than CBT alone in the improvement of daytime dysfunction. Therefore, the combination of EA and CBT is more effective in treating chronic insomnia than EA or CBT treatment alone.

Although this study found that the curative effect of the combined EA/CBT therapy was longer in duration than that of the EA therapy, the curative effect of EA versus CBT alone was similar in comparison during the follow-up period. This indicates that the long-term efficacy of the combined EA/CBT therapy is superior to that of EA therapy alone. It can be seen from the time-dependent statistical processing and the dynamic trend graph of each evaluation scale score that the EA group had a faster decline in outcome scores during the first two weeks of treatment, which gradually entered a plateau as the treatment time prolonged, and there was a slight rebound after the conclusion of treatment. This may be related to the repeated stimulation of fixed acupoints, resulting in acupoint fatigue. In contrast, the CBT group had the opposite trend, with the decline trend being slower in the early stage of treatment, but its efficacy trend was equivalent to the EA group with the extension of treatment time, which may be related to the treatment characteristics of CBT and the design of the group activities. When using EA and CBT at the same time, the superposition effect does not reflect the increase in the degree of curative effect but is reflected in the increase of persistent efficacy.

In summary, in the early stage of treatment, EA can reduce the use of sedative hypnotic drugs and increase the sleep time more quickly, while the CBT group focuses on improving the nighttime awakening and difficulty falling asleep. When the two treatments were combined, it was found that the improvement of overall sleep quality and sleep latency during the follow-up period increased.

This study found that, during the treatment period, the EA group significantly improved their daytime function and reduced the degree of lethargy compared with the CBT group. However, during the follow-up period, the EA group and the CBT group had little difference in the degree of lethargy. The daytime sleepiness of the EA/CBT combined group was significantly lower than that of the single treatment groups. The early mild to moderate sleep deficit of CBT sleep restriction therapy is an important cause of daytime dysfunction. Most of the acupuncture treatments do not have this problem; EA improves the daytime function of patients in the early stage of treatment and reduces the use of sedative hypnotic drugs more quickly. Reducing the daytime residue of sedative hypnotic drugs can also help improve daytime function. Therefore, the combined EA/CBT treatment may provide the respective advantages of the two treatments while offsetting the disadvantages of EA or CBT alone, thus leading to better clinical outcomes.

The results of this study indicate that all three treatment options can effectively improve anxiety and depression, but after the treatment, the combined EA/CBT group had better outcomes in this regard, which suggests that the combination of the two nondrug interventions (EA as Traditional Chinese Medicine (TCM) and CBT as modern psychology) has a synergistic effect on improving mood and can better solve the emotional problems associated with chronic insomnia. Although CBT-I does not have the specific aim to improve other psychiatric symptoms such as depression or anxiety, many studies on insomnia comorbidities and mental illness suggest that it still has a good effect on mood [32, 33]. The CBT mechanism may reduce the functional connection between the thalamus and parietal cortex, the putamen and motor cortex, and the amygdala and lingual gyrus and enhance the functional connection between the caudate nucleus and supramarginal gyrus, the globus pallidus and orbital frontal cortex, and the hippocampus and frontal parietal gyrus [34]. The specific acupoints used can also adjust the mood, which has been confirmed by many modern studies, and the single-point studies of DU-20, HT-7, and BL-62, which were selected in this study, have been confirmed to have the effect of adjusting emotions and are widely used in the clinical treatment of mental and emotional illness [35, 36].

### 4.3. TCM Theory Basis for Spirit-Regulating EA Combined with CBT Therapy in Chronic Insomnia

In the 1970s, modern medicine proposed a new bio-psychological-social model, which is highly consistent with the etiology and pathogenesis of chronic insomnia. As early as the period of Huangdi Neijing, TCM put forward the holistic view of disease treatment, which emphasized the unity of form and spirit. It not only advocated the harmony between human and nature, but also emphasized the complementarity of physical basis and functional activities of the organism. There are many similarities between the TCM “form and spirit” theory and the modern psychosomatic theory. The TCM “form and spirit” theory pays attention to the combination of form and spirit, emphasizing the interaction between psychological factors and biological factors, and is therefore suitable to guide the treatment of chronic insomnia. Acupuncture, according to the “form and spirit” theory, emphasizes regulating the spirit. In the course of treatment, the thought of “calming, keeping, and regulating the spirit” runs through the mind, which has the effect of treating mind and body together. The mechanism of acupuncture is achieved through acting on the brain center and autonomic nervous system, which is closely related to the pathogenesis of chronic insomnia. CBT adjusts cognition through psychotherapy, adjusting the rhythm of work and rest through behavioral therapy, and at the same time teaches relaxation therapy, striving to treat mind and body together, giving consideration to being both spirit-governing and body-preserving.

To sum up, the form and spirit theory provides the macroscopical theory guidance for chronic insomnia. Acupuncture is inspired by this theory and attaches importance to regulating the spirit. CBT, though not derived from the “form and spirit” theory, is highly similar to TCM in its understanding of chronic insomnia. Both EA and CBT can regulate the spirit. The efficacy of CBT to treat insomnia is established in the literature, while acupuncture lacks clinical evidence to support its efficacy in the treatment of insomnia. In addition, the combination of EA and CBT may prove to be an effective therapy modality to clinically treat chronic insomnia.

### 4.4. Limitations

This study was conducted at a single center. Study participants were recruited from a large, urban Chinese medical hospital. Therefore, selection bias may be present. The definition of chronic insomnia was not stratified as part of this study. The follow-up period for this study was only two weeks. A multicenter randomized clinical trial which includes chronic insomnia stratification and a follow-up period of at least 1 month will provide further evidence to support the use of various treatment modalities to treat insomnia.

This study found that acupuncture-controlled CBT can better improve daytime function. In the future, more objective multiple sleep latency tests (MSLT), pre-sleep arousal scales (PSAS), and hyperarousal scales (HAS) can be used for a more comprehensive evaluation.

This study lacks objective examinations such as polysomnography (PSG). In the future, we may consider using fMRI technology to explore the effects of acupuncture and CBT on different sleep-related brain areas.

Group therapy was adopted in this study, so there were time differences between the screening date and the treatment date for the first and last participants in each group. It was not possible to judge whether this affected the results of the study.

### 4.5. Summary

1. Electroacupuncture is effective in alleviating chronic insomnia and can improve sleep quality and emotional state at the same time. The overall efficacy is similar to that of CBT. We have adopted a standardized acupuncture program, with safe acupoint locations, which can be used as a suitable technique for treating chronic insomnia in clinical work.
2. The combined therapy of electroacupuncture and CBT has more advantages than that of the single therapy, which is reflected in its quicker improvement onset, longer lasting effect, better daytime function, and a more prominent improvement of mood. Interdisciplinary treatment can be beneficial to patients who suffer from chronic insomnia.
3. The mechanism of spirit-regulating electroacupuncture therapy is different from that of CBT. The combination can play a clinical synergistic role.

## Figures and Tables

**Figure 2:**
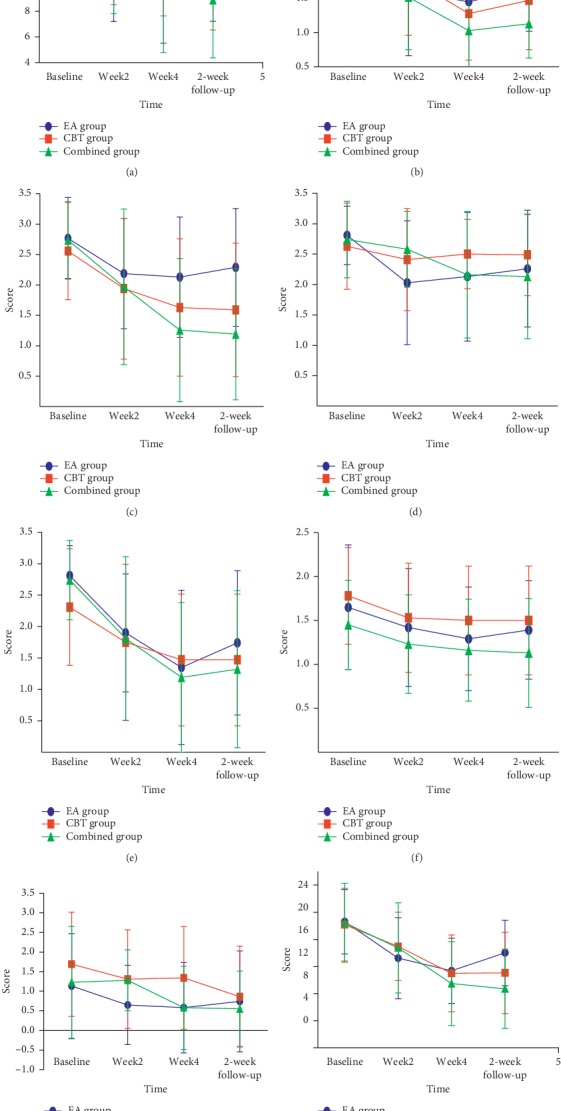
Change in PSQI total score, six subscale scores, and ISI score at different times. (a) PSQI total score. (b) Sleep quality factor. (c) Sleep latency factor. (d) Sleep persistence factor. (e) Sleep efficiency factor. (f) Sleep disorder factor. (g) Hypnotic drug factor. (h) ISI.

**Table 1:** Demographic and clinical characteristics (Mean ± SD).

Variables	EA (*n* = 31)	CBT (*n* = 32)	EA/CBT (*n* = 31)	X2/F/H value	*P* Value
Gender					
Male	3 (9.68%)	9 (28.13%)	6 (19.35%)	3.46	0.18
Female	28 (90.32%)	23 (71.88%)	25 (80.65%)		
Age, years	56.32 ± 9.01	49.41 ± 14.00	54.45 ± 12.10	2.85	0.063
Marital status					
Unmarried	0 (0%)	3 (9.38%)	4 (12.90%)		
Married	30 (96.77%)	24 (75.00%)	27 (87.10%)	3.46	0.18
Divorced	1 (3.23%)	2 (6.25%)	0 (0%)		
Widowed	0 (0%)	3 (9.38%)	0 (0%)		
Insomnia course				4.00	0.14
	72 (30–240)	42 (24–96)	60 (24–180)		
Education level				4.97	0.083
Primary school	1 (3.23%)	0 (0%)	0 (0%)		
Middle school	17 (54.84%)	11 (34.38%)	13 (41.94%)		
Bachelor's degree	13 (41.94%)	20 (62.5%)	18 (58.06%)		
Postgraduate	0 (0%)	1 (3.13%)	0 (0%)		
Sedative drug use (tablet)	4 (0–12)	0 (0–8)	0 (0–12)	1.01	0.60
HAM-D	11.35 ± 5.12	11.63 ± 5.73	10.65 ± 5.06	0.28	0.75
HAM-A	11.42 ± 4.23	11.81 ± 5.10	10.61 ± 4.37	0.56	0.57
DBAS-16	88.52 ± 29.76	93.5 ± 27.34	96.23 ± 31.68	0.54	0.58
PSQI	16.00 ± 2.56	16.09 ± 3.86	15.84 ± 2.68	1.06	0.59
ISI	18.65 ± 4.67	18.63 ± 5.53	18.45 ± 5.80	0.17	0.92
ESS	6.23 ± 5.38	4.74 ± 0.80	5.77 ± 5.19	0.28	0.87

**Table 2:** DBAS-16 before and after treatment in the three groups ($\bar{x}\pm s$).

	Baseline	Week four
EA (*n* = 31)	88.52 ± 29.76	85.61 ± 27.98^Δ^^*∗*^
CBT (*n* = 32)	93.5 ± 27.34	67.5 ± 27.18
EA/CBT (*n* = 31)	96.23 ± 31.68	67.19 ± 33.03

Note:^*∗*^EA group versus EA/CBT group, *P* < 0.05; ^Δ^EA group versus CBT group, *P* < 0.05.

**Table 3:** HAM-A scores before and after treatment in the three groups ($\bar{x}\pm s$).

	Baseline	Week four
EA (*n* = 31)	11.42 ± 4.23	9.52 ± 4.02^*∗*^
CBT (*n* = 32)	11.81 ± 5.10	9.41 ± 4.73^Δ^
EA/CBT (*n* = 31)	10.61 ± 4.37	6.06 ± 5.27

Note:^*∗*^EA group versus EA/CBT group, *P* < 0.05; ^Δ^CBT group versus EA/CBT group, *P* < 0.05.

**Table 4:** Difference analysis of HAM-D before and after treatment in three groups ($\bar{x}\pm s$).

	Baseline	Week 4
EA (*n* = 31)	11.35 ± 5.12	9.71 ± 5.50^*∗*^
CBT (*n* = 32)	11.63 ± 5.73	9.56 ± 6.24^Δ^
EA/CBT (*n* = 31)	10.65 ± 5.06	5.29 ± 4.42

Note:^*∗*^EA group versus EA/CBT group, *P* < 0.05; ^Δ^CBT group versus EA/CBT *P* < 0.05.

## Data Availability

All the data used to support the findings of this study are included within the supplementary information file, and they are available from the corresponding author upon request.

## Supplementary Materials

Supplementary Materials Document 1: Gender (1-male; 2-female) Document 2: Age Document 3: Marital status (0-unmarried; 1-married; 2-divorced; 3-widowed) Document4: education (1-Primary school; 2-Middle school; 3-Undergraduate; 4-Graduate student) Document 5: duration Document 6: Sedative drug use Document 7: HAM-D total score-before treatment Document 8: HAM-A total score-before treatment Document 9: DBAS-16-before treatment Document 10: PSQI total score-before treatment Document 11: ISI-before treatment Document 12: ESS-before treatment Document 13: PSQI total score-baseline; the 2nd week; the 4th week; the 2nd week of follow-up Document 14: Sleep quality factor-baseline; the 2nd week; the 4th week; the 2nd week of follow-up Document 15: Sleep latency factor-baseline; the 2nd week; the 4th week; the 2nd week of follow-up Document 16: Sleep persistence factor-baseline; the 2nd week; the 4th week; the 2nd week of follow-up Document 17: Sleep efficiency factor-baseline; the 2nd week; the 4th week; the 2nd week of follow-up Document 18: Sleep disorder factor-baseline; the 2nd week; the 4th week; the 2nd week of follow-up Document 19: Hypnotic drug factor-baseline; the 2nd week; the 4th week; the 2nd week of follow-up Document 20: ISI-baseline; the 2nd week; the 4th week; the 2nd week of follow-up Document 21: Daytime dysfunction factor-baseline; the 2nd week; the 4th week; the 2nd week of follow-up Document 22: ESS-baseline; the 2nd week; the 4th week; the 2nd week of follow-up Document 23: DBAS-16 before and after treatment Document 24: HAM-A before and after treatment.
