# Supplementary material for: Effects of Electroacupuncture Therapy and Cognitive Behavioral Therapy in Chronic Insomnia: A Randomized Controlled Study
Source: Evid Based Complement Alternat Med. 2020 Mar 19;2020:5630130. doi: 10.1155/2020/5630130 (PMC7106874; doi:10.1155/2020/5630130)
Supplement: Supplementary Materials — Document 1: Gender (1-male; 2-female) Document 2: Age Document 3: Marital status (0-unmarried; 1-married; 2-divorced; 3-widowed) Document4: education (1-Primary school; 2-Middle school; 3-Undergraduate; 4-Graduate student) Document 5: duration Document 6: Sedative drug use Document 7: HAM-D total score-before treatment Document 8: HAM-A total score-before treatment Document 9: DBAS-16-before treatment Document 10: PSQI total score-before treatment Document 11: ISI-before treatment Document 12: ESS-before treatment Document 13: PSQI total score-baseline; the 2nd week; the 4th week; the 2nd week of follow-up Document 14: Sleep quality factor-baseline; the 2nd week; the 4th week; the 2nd week of follow-up Document 15: Sleep latency factor-baseline; the 2nd week; the 4th week; the 2nd week of follow-up Document 16: Sleep persistence factor-baseline; the 2nd week; the 4th week; the 2nd week of follow-up Document 17: Sleep efficiency factor-baseline; the 2nd week; the 4th week; the 2nd week of follow-up Document 18: Sleep disorder factor-baseline; the 2nd week; the 4th week; the 2nd week of follow-up Document 19: Hypnotic drug factor-baseline; the 2nd week; the 4th week; the 2nd week of follow-up Document 20: ISI-baseline; the 2nd week; the 4th week; the 2nd week of follow-up Document 21: Daytime dysfunction factor-baseline; the 2nd week; the 4th week; the 2nd week of follow-up Document 22: ESS-baseline; the 2nd week; the 4th week; the 2nd week of follow-up Document 23: DBAS-16 before and after treatment Document 24: HAM-A before and after treatment. [file 5630130.f1.zip › 5630130.f1/data/Introduction.docx]

Introductions

The data in the manuscript are listed in Excel sheets and named accordingly. Group 1 in all excel sheets represents the electroacupuncture group, Group 2 represents the CBT group, Group 3 represents the combination group.
